# Cilia-associated wound repair mediated by IFT88 in retinal pigment epithelium

**DOI:** 10.1038/s41598-023-35099-3

**Published:** 2023-05-21

**Authors:** Ke Ning, Mohajeet B. Bhuckory, Chien-Hui Lo, Brent E. Sendayen, Tia J. Kowal, Ming Chen, Ruchi Bansal, Kun-Che Chang, Douglas Vollrath, Nicolas F. Berbari, Vinit B. Mahajan, Yang Hu, Yang Sun

**Affiliations:** 1 Department of Ophthalmology, Stanford University School of Medicine, 1651 Page Mill Road, Rm 2220, Palo Alto, CA 94304 USA; 2 Palo Alto Veterans Administration, Palo Alto, CA USA; 3 Department of Genetics, Stanford University School of Medicine, Palo Alto, CA USA; 4 Department of Biology, Indiana University-Purdue University Indianapolis, Indianapolis, IN USA; 5 Department of Ophthalmology, University of Pittsburgh School of Medicine, Pittsburgh, PA USA

**Keywords:** Immunochemistry, Biochemistry, Cell biology, Molecular biology, Diseases, Medical research, Molecular medicine, Pathogenesis

## Abstract

Primary cilia are conserved organelles that integrate extracellular cues into intracellular signals and are critical for diverse processes, including cellular development and repair responses. Deficits in ciliary function cause multisystemic human diseases known as ciliopathies. In the eye, atrophy of the retinal pigment epithelium (RPE) is a common feature of many ciliopathies. However, the roles of RPE cilia in vivo remain poorly understood. In this study, we first found that mouse RPE cells only transiently form primary cilia. We then examined the RPE in the mouse model of Bardet-Biedl Syndrome 4 (BBS4), a ciliopathy associated with retinal degeneration in humans, and found that ciliation in BBS4 mutant RPE cells is disrupted early during development. Next, using a laser-induced injury model in vivo, we found that primary cilia in RPE reassemble in response to laser injury during RPE wound healing and then rapidly disassemble after the repair is completed. Finally, we demonstrated that RPE-specific depletion of primary cilia in a conditional mouse model of cilia loss promoted wound healing and enhanced cell proliferation. In summary, our data suggest that RPE cilia contribute to both retinal development and repair and provide insights into potential therapeutic targets for more common RPE degenerative diseases.

## Introduction

Retinal pigment epithelium (RPE) is a polarized monolayer of cells essential for photoreceptor development and visual function because its interaction with photoreceptors is critical for retinal maintenance. Consequently, RPE dysfunction is related to many diseases including macular degeneration^1–5^. Age-related macular degeneration (AMD) is a leading cause of irreversible blindness in the elderly population^6–8^. Despite considerable advances in the treatment of wet AMD, there are no effective strategies for treating dry AMD, which includes 90% of all diagnosed cases^9–11^. Loss of RPE is postulated to be a major cause of AMD^12–15^. Understanding the potential for endogenous RPE regeneration could reveal novel potential therapeutic strategies for diseases like dry AMD^16–19^.

The primary cilium is a solitary microtubule-based structure present in almost all post-mitotic mammalian cell types^20–26^. This small cellular appendage can detect changes in the external environment and transduce these cues into intracellular signaling cascades. Because they are essential for normal embryogenesis, disruption of primary cilia causes developmental malformations in nearly all organ systems^20,27–32^. Primary cilia also play important roles in post-developmental processes such as cell proliferation and wound repair^20,33–39^. Mutations in genes necessary for cilia formation and function cause a spectrum of human diseases, collectively termed ciliopathies, which present as clinical disorders such as polycystic kidney disease, obesity, retinal degeneration, and RPE atrophy^40–45^. IFT88 is part of the intraflagellar transport complex that is required for ciliogenesis^46^. The prototypical ciliopathy Bardet-Biedl syndrome (BBS) results from mutations in BBS genes. Products for several BBS genes for the BBSome, an important multimeric protein complex critical for regulating the signaling composition of the ciliary compartment^47–52^. The BBSome member, BBS4, plays multiple roles in cell proliferation and cilia maintenance^53,54^.

Growing evidence supports a role for cilia in coordinating cell proliferation and migration, which when dysfunctional, can lead to defects in wound healing^33,34,37,55–59^. Because the cilium points in the migratory direction, it is thought that primary cilia may participate in the cyclic processes in directional cell migration during tissue repair^37,58^. For example, in skin cells, primary cilia play a major role in controlling proliferation in wound healing^60^. It is widely known that defective cilia enhance cell proliferation during the pathogenesis of diverse disorders^33,56,61^. In another example, deletion of a different intraflagellar transport gene, IFT20, in mouse kidney collecting duct cells results in proliferation and cyst development that lead to cystic kidney disease^62^.

Immortalized RPE cell lines have been used extensively as a model system to study cilia protein complexes and ciliogenesis ^22,38^. However, the roles of RPE cilia in vivo remained largely unknown. Recently, cilia were discovered to be required for RPE maturation^63,64^. Because rodent RPE cilia are resorbed after maturation^64,65^, it remains unclear whether cilia renewal contributes to repair activities and regeneration of RPE in adulthood.

Here, we investigated the role of primary cilia in the proliferative and reparative responses of RPE of mice to targeted laser ablation. We show that BBS4 plays a significant role in RPE ciliogenesis in vivo. Utilizing transgenic mice and immunofluorescence technology, we also provide evidence that primary cilia of adult mouse RPE are reassembled in response to laser-induced injury during wound healing. Furthermore, we characterize the function of primary cilia, by utilizing a conditional allele of *Ift88* to ablate cilia and their potential formation in postnatal RPE. We demonstrated that cilia removal promotes tissue repair by enhancing cell proliferation.

## Results

### Primary cilia disassembly during development in albino mouse RPE

**Figure 1:**
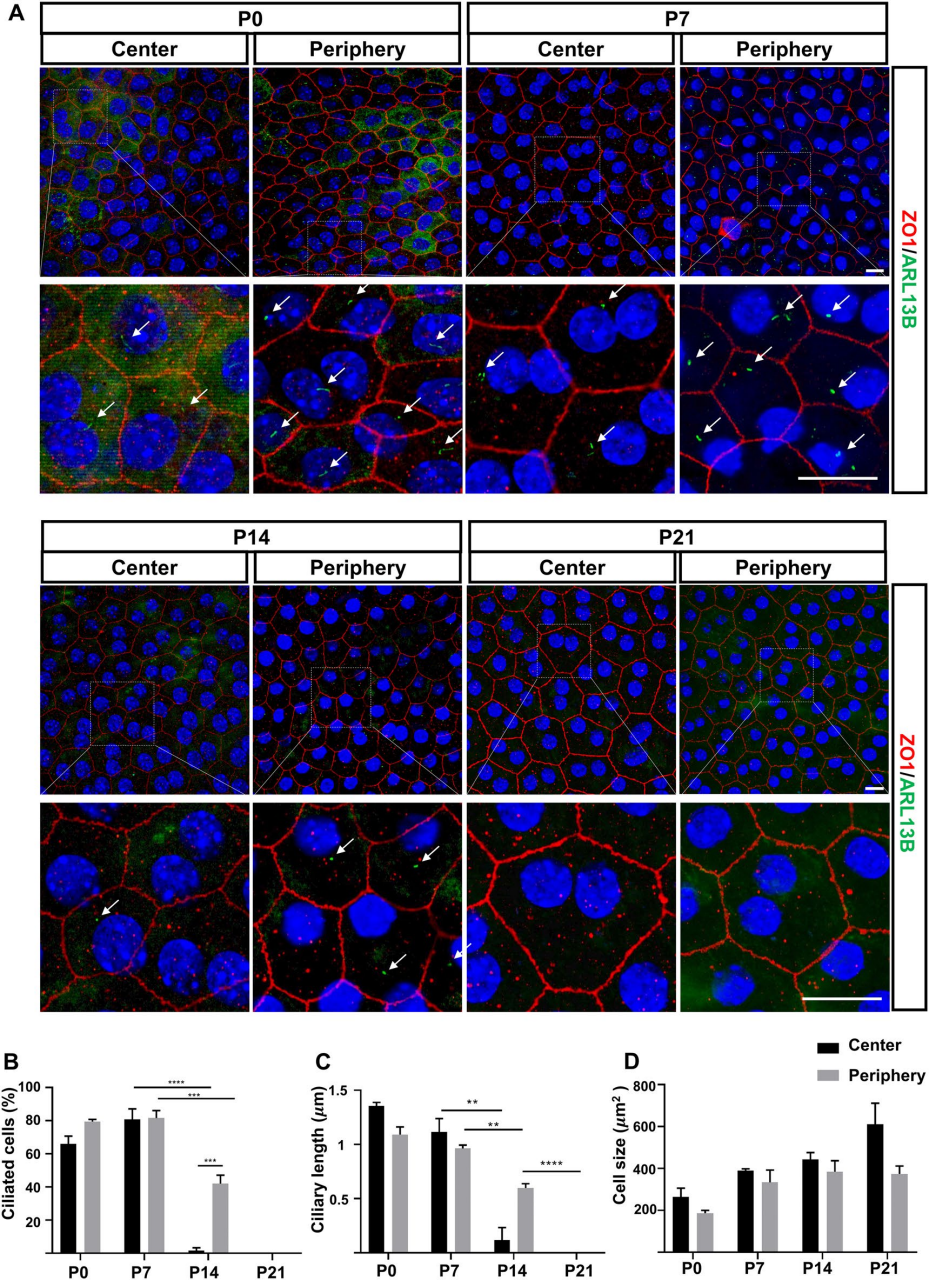
Primary cilia disassembly during development in albino mouse RPE. Primary cilia assembly and disassembly are dynamically regulated during the development of RPE in CD1 albino mice. (**A**) Confocal images from flat mount retina samples at different postnatal ages showing the peripheral or central areas of RPE. ZO-1 staining cell junctions (red), Arl13b is a ciliary marker (green), nuclei (blue). Magnifications of each corresponding image are shown. Scale bars: small 20 μm; magnified images 20 μm. Arrows indicate cilia. (**B**) Quantification of ciliation in RPE during development. P0 n = 50 cells, P7 n = 50 cells, P14 n = 50 cells, P21 n = 50 cells. Number of eyes:3 or 4 per timepoint. (**C**) Quantification of cilium length (**D**) Quantification of cell area. Statistical analyses were performed using Student’s t-test, p < 0.05 was considered statistically significant.

Previous studies reported that primary cilia of rodent RPE disassemble after retinal maturation^64,65^. However, the timing of cilia disassembly is largely different in rats compared to pigmented C57BL/6 mice^64,65^. While more than 60% of RPE cells contain cilia in rats on the day after birth post-natal day 1 (P1), there are only a few ciliated RPE found in C57BL6 mice. We were interested in learning if this was due to a species difference between mice and rats. To further investigate the extent to which cilia are resorbed in mouse RPE in another strain, we analyzed the RPE of albino CD1 mice in flatmount retina preparations from P0 to P21 (Fig. 1A). Primary cilia were identified by immunofluorescence for Arl13b, a commonly used cilia membrane marker^57,66^. Tight junctions were visualized by immunofluorescence for ZO-1^67^. RPE cells with primary cilia were abundant at post-natal day 0 (P0), but their frequency was significantly decreased in 3-week-old animals (P21), when the retina was considered to be largely mature. Interestingly, the percentage of ciliated cells in the central area (near the optic nerve) was greatest at P7 (approximately 80%), whereas only a few ciliated RPE were detected at P7 in pigmented mice^64^. By P14, the reduction of ciliated cells was significantly greater in central RPE than in peripheral RPE. Primary cilia were undetectable in both peripheral and central RPE at P21, suggesting that most RPE cells had lost their cilia or that the length of their cilia was reduced or that they no longer were Arl14b positive (Fig. 1B). Changes in Arl13b stained ciliary length accompanied cilia resorption: mean length decreased from 1.4 μm at P0 to 0.2 μm at P14 in the central area, and from 1.1 μm at P0 to 0.6 μm at P14 in the peripheral area (Fig. 1C). Analysis of RPE cell areas at different developmental stages showed a trend toward a progressive increase in cell size at both central and peripheral areas (Fig. 1D). Interestingly, we observed a group of bi-ciliated RPEs during developmental stages (Fig. S1). At P0, 1.62 ± 1.44% of ciliated RPE were bi-ciliated in the periphery area, whereas 10.26 ± 5.43% in the central area. At P7, 12.11 ± 3.50% of ciliated RPE were bi-ciliated in the periphery area, whereas 13.58 ± 6.99% in the central area. The RPEs that were bi-ciliated were either mononucleated or binucleated. Taken together, these results showed that the timing of resorption of primary cilia in the CD1 mice is different from C57CL6. In addition, cilia disassembly varies in the central and peripheral areas in CD1 mice. Starting at P0 in the peripheral area and at P7 in the central area, cilia progressively disassemble in the RPE of these mice and appear to be nearly absent by P21.

### Loss of BBS4 affects RPE cilia

**Figure 2:**
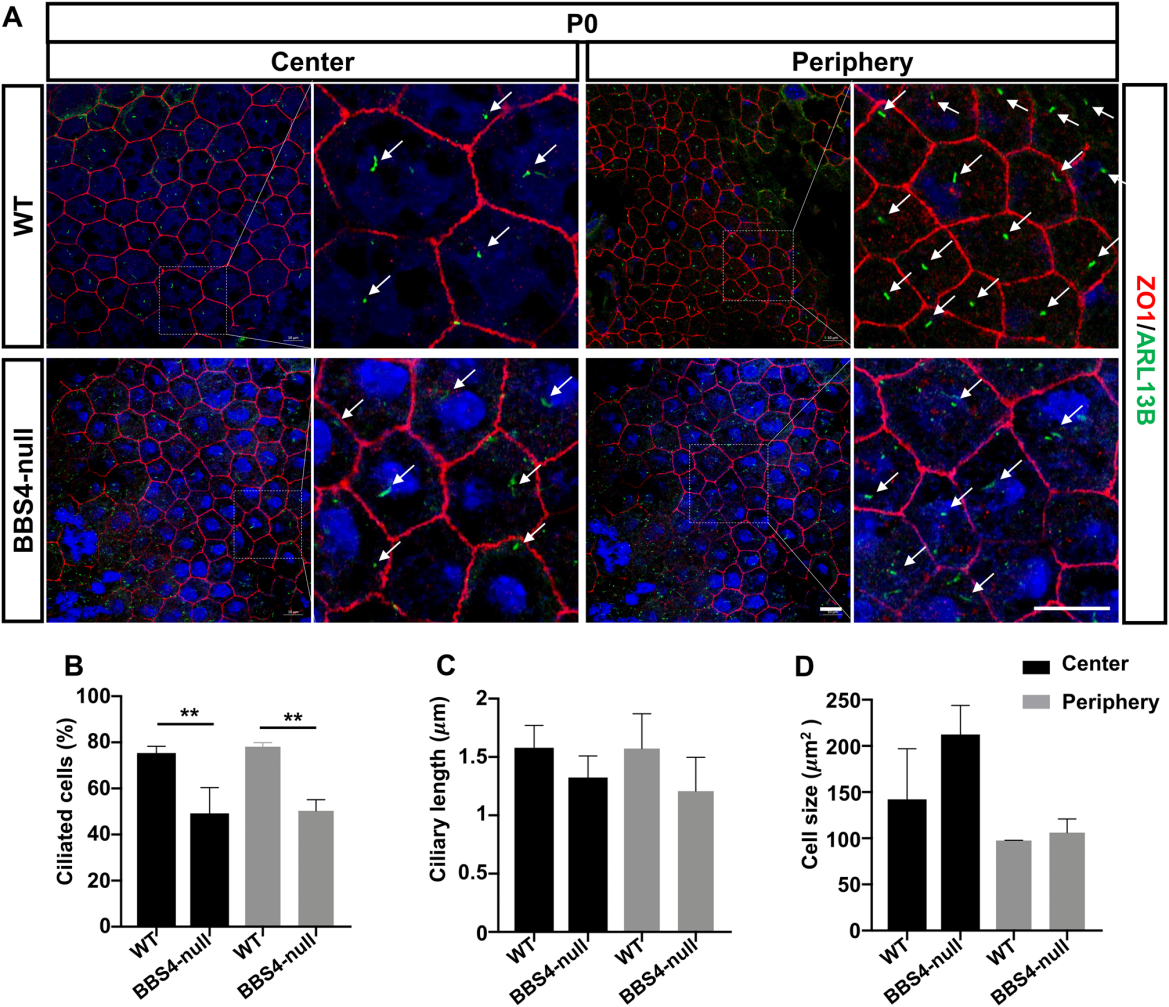
Loss of BBS4 affects ciliation in RPE in vivo. BBS4 knockout alters RPE ciliation in vivo. (**A**) Representative immunofluorescence images of P0 mouse RPE flatmounts from both peripheral and central areas of WT and BBS4-null littermates, labeled with antibodies for primary cilia and cell junctions (Arl13b in green and ZO-1 in red) Scale bars: small 20 μm; magnified images 20 μm. Arrows indicate cilia. (**B**) Quantification of ciliation in RPE during development. On average approximately 200–300 cells at the center or at the periphery of the RPE were counted for each age group. n = 3 eyes. (**C**) Quantification of ciliary length in RPE during development. (**D**) Quantification of cell size in RPE during development. Scale bars: 20 μm. n = 1. Statistical analyses in (**B**)–(**D**) were performed using Student’s t-test, where P < 0.05 was considered statistically significant.

BBS4 knockout mice recapitulate many of the human ciliopathy phenotypes, including retinal degeneration^68^. To investigate whether BBS4 plays a role in RPE ciliogenesis and maturation and could contribute to their retinal degeneration, we analyzed RPE in flatmounts from germline Bbs4-null mice at P0 and in age-matched controls using the same approach that we took for our CD1 albino analysis (Fig. 2A). Our results showed that ciliation levels were significantly decreased in Bbs4-null RPE compared with control cells. For the central area, the ciliation of RPE flatmounts in Bbs4-null mice was 53.8% compared to 74.9% in the age-matched control group. For the peripheral area, the ciliation of RPE flatmounts in Bbs4-null mice was 51.2% compared to 78.1% in the wildtype control (Fig. 2B). Consistent with the ciliation results, ciliary length was also moderately shorter in Bbs4-null RPE compared to controls in both central and peripheral areas (Fig. 2C) although the differences were not statistically significant. Mean ciliary length in RPE flatmounts was 1.3 μm in the central area of Bb4-null mice and 1.6 μm in the wildtype control; 1.2 μm in the peripheral area in Bbs-null RPE and 1.6 μm in the wildtype control. In addition, there was a trend toward increased cell size in the central area of the mutant group, whereas cell size in the peripheral area remained unchanged (Fig. 2D). In addition, the general ZO-1 expression level revealed no significant difference between control and Bbs4-null mice.

### Defective primary cilia in RPE from age-related macular degeneration (AMD) patient

**Figure 3:**
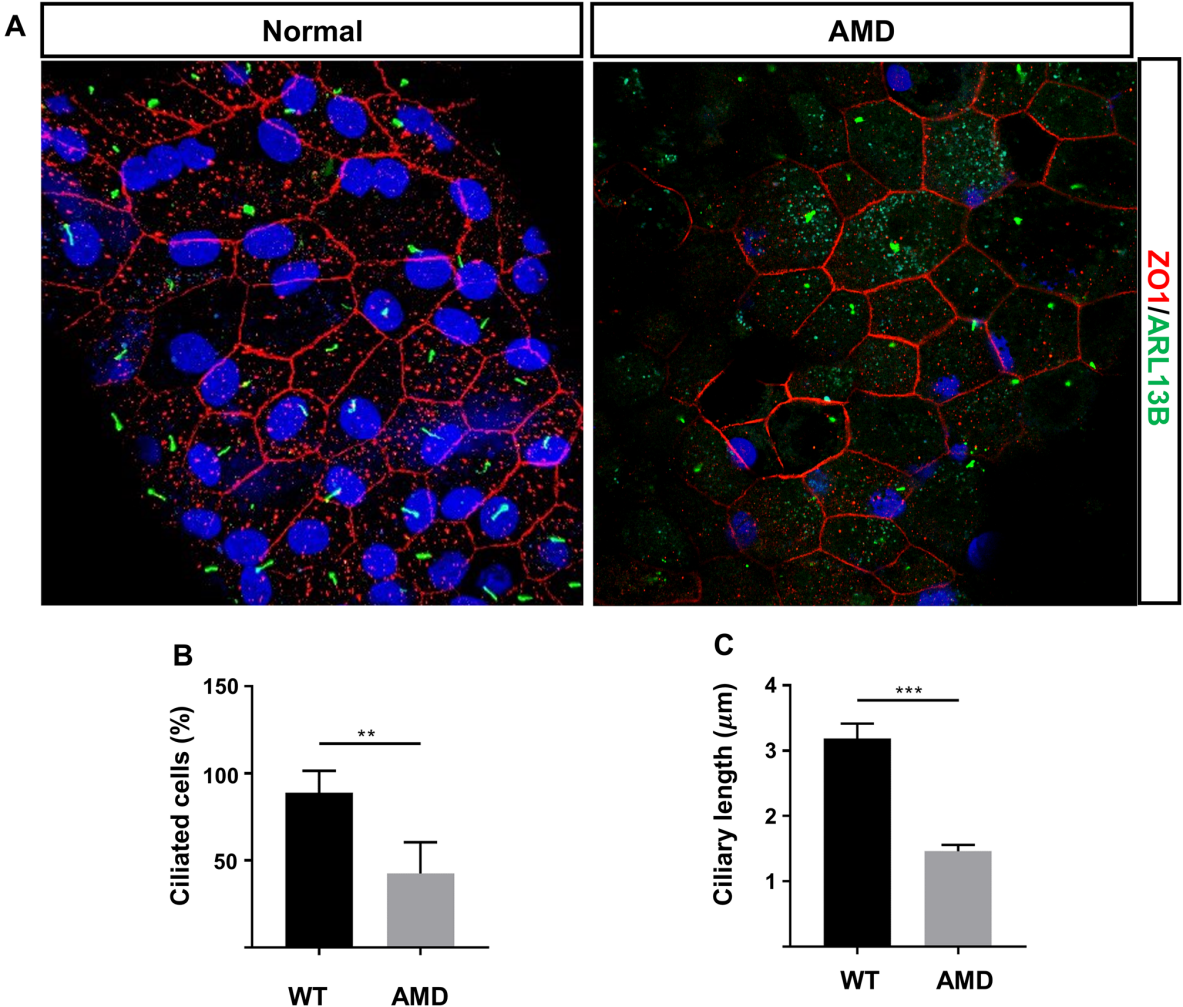
Defective primary cilia in RPE from AMD patient. (**A**) Confocal images stained for Arl13b (green) and ZO-1 (red) of healthy human RPE and AMD patient. (**B**) Quantification of ciliation in RPE (4–5 individual 63 × confocal images were imaged/counted from the periphery area of each human RPE sample. 223 × 223 µm^2^/image). (**C**) Quantification of ciliary length in RPE. Scale bars: 10 μm. n = 1. Statistical analyses in (**B**)–(**D**) were performed using Student’s t-test, where p < 0.05 was considered statistically significant.

We further investigated the ciliary profile in adult human RPE tissue. Before performing immunohistochemistry, we used the iDiSCO clearing method, which renders intact tissue transparent^69^. The results revealed that adult human RPE formed primary cilia, as confirmed by ARL13B labeling, and that cilia were present in almost all cells across the monolayer (Fig. 3). The average length of primary cilia was 3.2 μm. In addition, we found a significant decrease in both ciliation and ciliary length in RPE from AMD patient (n = 1) compared with healthy RPE. Together, these results showed that human RPE, unlike the macula-free murine counterpart, forms primary cilia in adults, suggesting that cilia play roles in the human RPE that persist after development is complete and that they may be defective in human disease conditions, such as macular degeneration.

### Hypopigmented and reduced RPE65 expression in RPE conditional cilia loss mice

**Figure 4:**
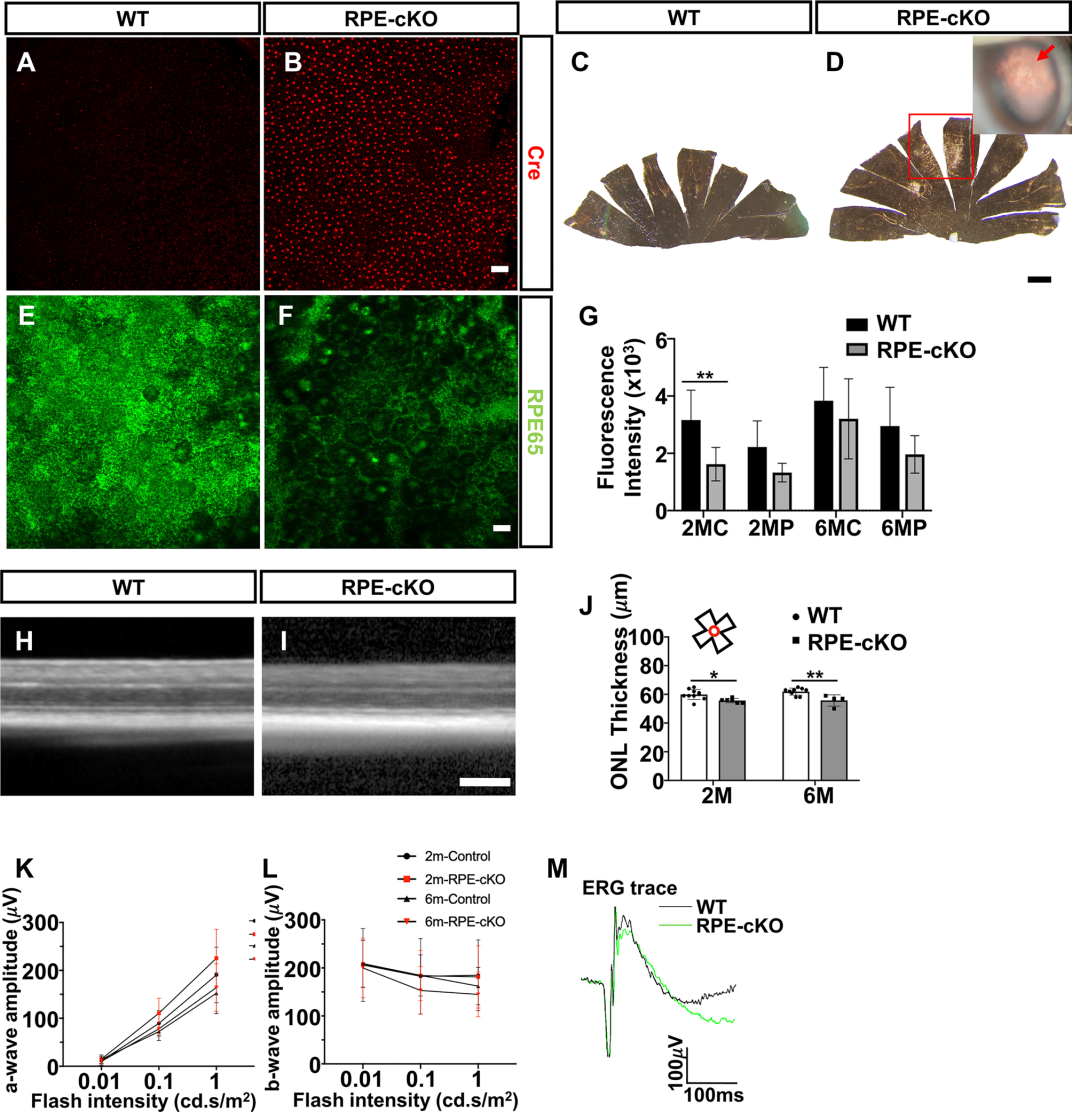
Hypopigmentation and loss of RPE65 upon conditional cilia loss in mice. Assessing the impact of conditional cilia loss on RPE, retina and visual function. (**A**, **B**) RPE flatmounts stained with Cre antibody (red) revealing Cre expression in RPE-cKO group. (**C**, **D**) RPE flatmount from RPE-cKO mice showing regional hypopigmentation in the peripheral RPE indicated with a red box. Scale bars: 300 μm. (**E**, **F**) RPE65 staining (green) in RPE flatmounts in both knockout and control groups at 2-months-old. Graph shows quantitative flourescence levels for RPE65 staining over time. (**H**–**J**) SD-OCT images of controls and RPE-cKO mouse retinas from 2-months-old to 6-months-old. Measurement of outer nuclear layer (ONL) thickness obtained from both timepoints. (**K**–**M**) Electroretinogram (ERG) recordings for RPE-cKO and control mice. Dark-adapted ERG showing the rod photoreceptor function. ONL; outer nuclear layer, RGL; retinal ganglion cell layer, INL; inner nuclear layer. n = 3 animals in 2-month RPE-cKO group; n = 5 animals in 2-month control group; n = 5 animals in 6-month RPE-cKO group; n = 3 animals in 6-month control. Statistical analyses were performed using one-way ANOVA, p < 0.05 was considered statistically significant. Scale bars: 20 μm.

To understand whether ciliary loss can impact postnatal RPE in vivo, we generated transgenic mice with RPE-selective postnatal loss of primary cilia (RPE-cKO mice). We combined a conditional (floxed) allele of the intraflagellar transport protein (Ift88), which is essential for cilia formation and maintenance, with a Cre allele-driven by the BEST1 promoter to drive RPE-specific cilia loss. The BEST1-Cre transgene drives ocular Cre expression that begins postnatally at about P10^70,71^. The mosaic expression of the BEST1-Cre in the RPE allowed us to simultaneously assess the impact of cilia loss on RPE and on control neighboring cells in the retina. We performed genotyping and examined Cre expression by immunostaining on RPE flatmounts from every mouse, then focused on mice with 90% expression for further analysis (Fig. 4A,B). We observed patchy pigment defects (regional hypopigmentation) in RPE-cKO mice on RPE flatmounts and by slit-lamp (Fig. 4C,D, Fig. S3). To investigate RPE maturation in RPE-cKO mice, we compared the fluorescence intensity of RPE65 on RPE flatmounts in RPE-cKO and control mice. We analyzed both the central area and peripheral area (Fig. 4E–G). We found a significant decrease in RPE65 expression in RPE-cKO mice compared to the control group. Next, we compared the expression of both ezrin, a polarity marker for RPE, and ZO-1 in RPE-cKO and control mice, but found no differences (Fig. S2). To assess the photoreceptor morphology and function in RPE-cKO mice, we evaluated the thickness of the photoreceptor layer by OCT imaging and photoreceptor electrical response by ERG in 2- and 6-months old mice (Fig. 4H–M, Fig. S2). Neither scotopic (dark-adaptation) nor photopic (light-adaptation) ERG responses showed significant differences of b- or a-wave amplitudes between RPE-cKO mice and controls at either 2- or 6-months of age. These data suggested that RPEs are mildly compromised, but not severely enough to damage photoreceptor activity.

### Primary cilia reassembly in adult RPE during repair

**Figure 5:**
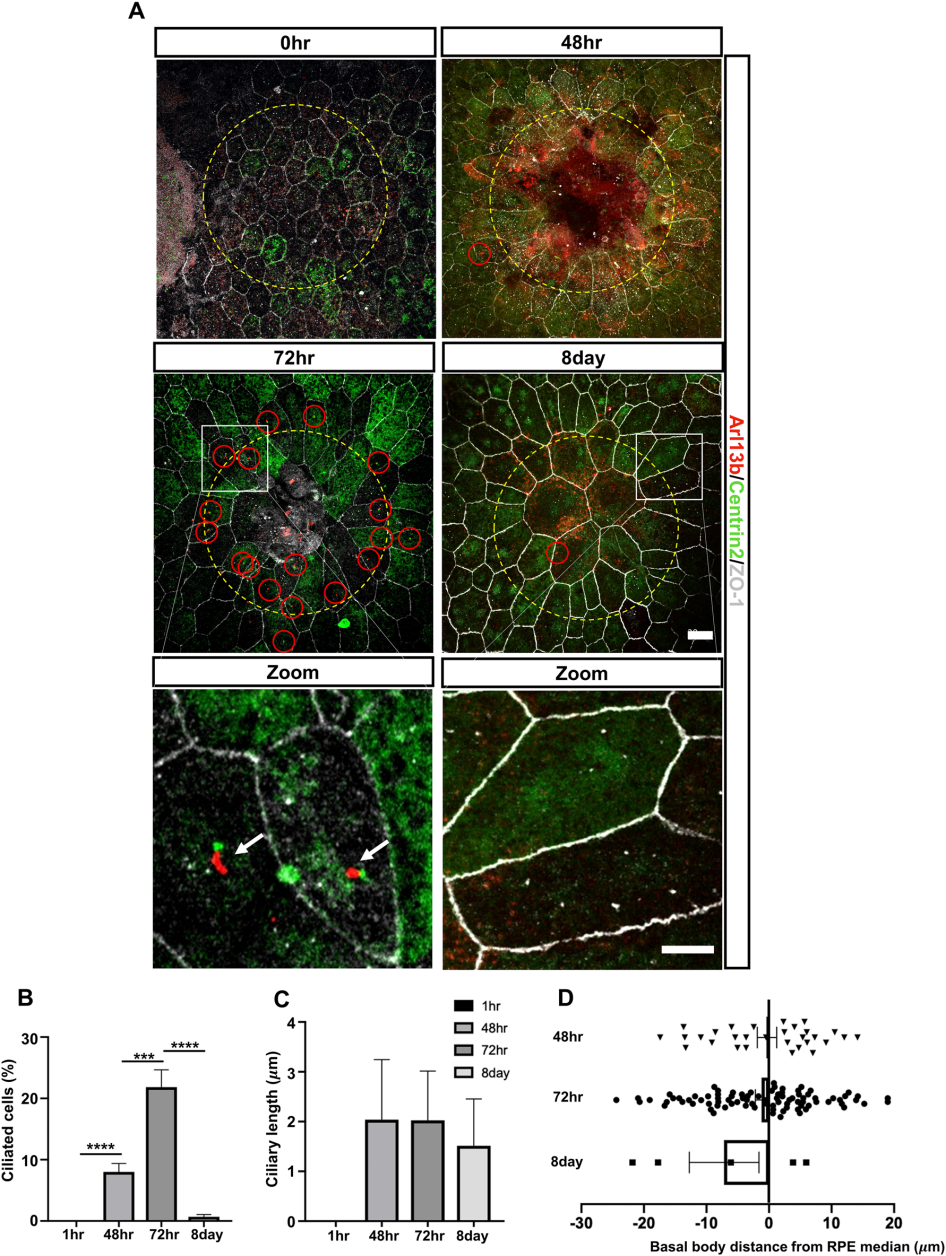
Primary cilia reassembly in adult RPE during repair. (**A**) Representative immunofluorescence images showing wound area of RPE flatmounts 0, 2, 3, 7 days after laser injury. Laser area (yellow dotted circles) with marked cilia (Arl13b in red and Centrin2 in green) and RPE border (white) shown on RPE flatmounts. At 1 h post laser, RPE near the laser area remain aciliated. Primary cilia (red circle) are detected on migrating and newly proliferated RPE near the laser area at 72 h and barely detected at day 8 post laser. Cell border is shown in white stained with ZO-1, Arl13b in red and Centrin2 in green. (**B**, **C**) Quantification of ciliation and ciliary length (n = 4). (**D**) Measurement between basal body location and cell medium. n = 3 animals per group. Scale bars: 20 μm. Statistical analyses were performed using One-way ANOVA, p < 0.05 was considered statistically significant.

In mouse corneal endothelium, primary cilia disassemble in adulthood but reassemble in response to injury-induced tissue repair^72^. These findings lead us to hypothesize that RPE cilia may also reform in response to injury and damage. We chose to test this idea, using a laser photocoagulation-induced injury approach which has been commonly used to investigate RPE wound healing in vivo^73–75^. We performed laser photocoagulation on double transgenic cilia and basal body reporter mice (Arl13b-mCherry; Centrin2-GFP), which would allow us to directly visualize cilia, centrioles and basal bodies in vivo using fluorescence microscopy^76–79^. The RPE/choroid layer was harvested at different timepoints after laser injury. RPE borders were labelled with ZO-1 and imaged as flatmounts. We found that the mouse RPE responded to injury by re-forming their cilia on migrating and newly proliferated cells during RPE wound healing and that primary cilia resorbed when repair appeared complete (Fig. 5A). Specifically, at 1 h post-laser injury, RPE cell borders were diminished within a clear edge of the laser spot pattern, indicating loss of RPE cells in this region. RPEs adjacent to the lasered area remained hexagonal in shape and non-ciliated. At 48 h after injury, primary cilia (Arl13b-mcherry reporter appeared adjacent to centrin-GFP basal body) first became detectable on enlarged and newly proliferating adjoining cells (small size cells). At 72 h after injury, most of the lasered area was covered by cells and additional ciliated cells. Interestingly, a few sporadic ciliated RPE have also detected on non-lasered areas 500 μm away from the laser site, suggesting that additional RPE cells were also responding even though their morphology remained unchanged. Primary cilia were barely detectable at day 7 after laser damage since the repair was completed. Measurements of ciliation and ciliary length during the repair process was measured and showed that ciliation was greatest at 72 h post laser (Fig. 5B,C). We further investigated whether basal body location related to migratory direction by measuring the distance between the basal body and cell median as described^72^. Our results showed no significant difference during wound healing, indicating that RPE cilia are not involved in cell pattern in repair (Fig. 5D). In summary, our data showed that primary cilia are reformed on adult RPE cells during wound healing, indicating that newly formed cilia may contribute to wound healing on mouse RPE in vivo.

### Defective cilia mediated by IFT88 promote RPE wound healing by controlling proliferation

**Figure 6:**
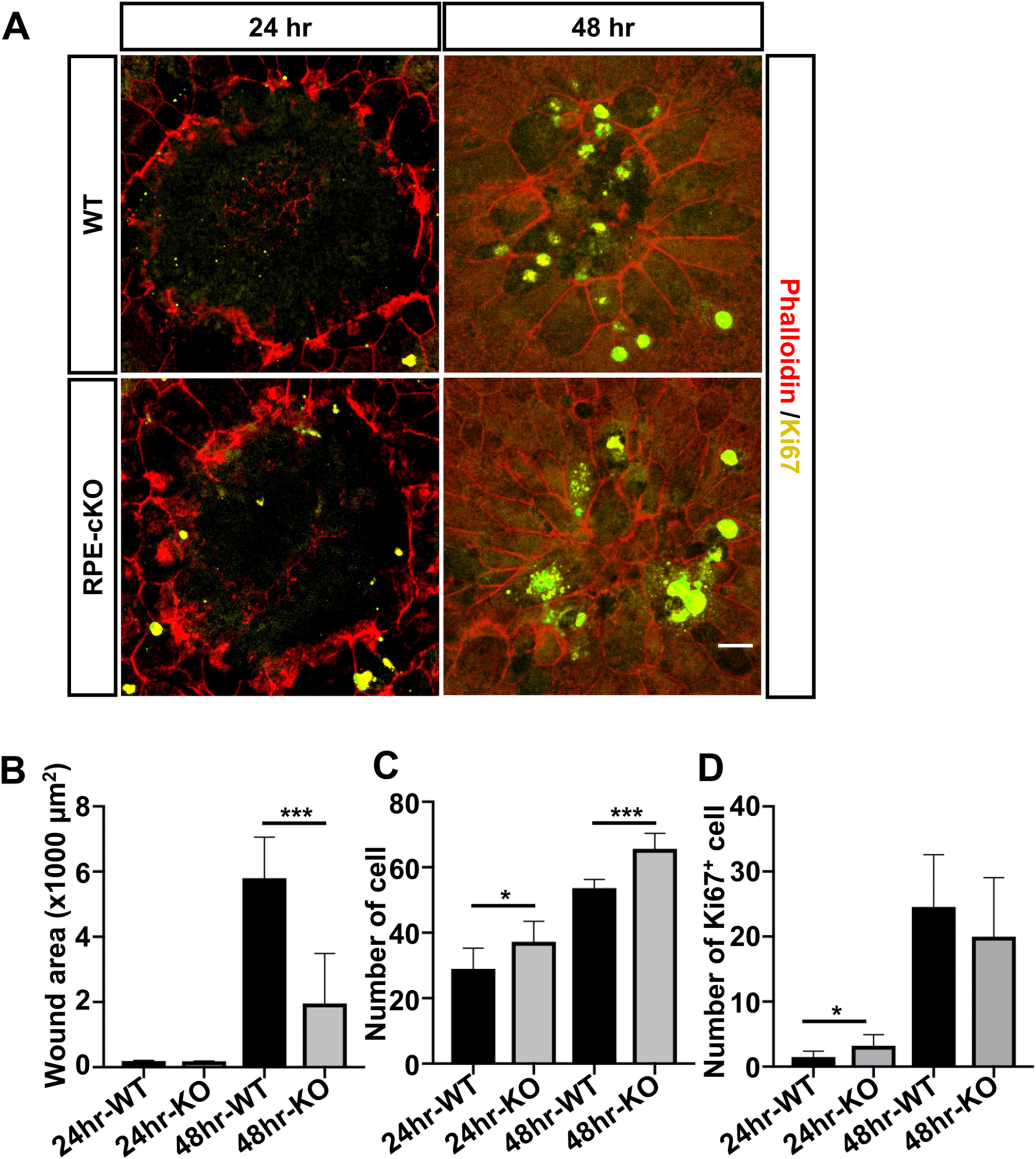
Defective cilia promote RPE wound healing by controlling proliferation. (**A**) Immunofluorescence analysis of phalloidin (red) and Ki67 (yellow) antibody in RPE flatmounts of RPE-cKO and control mice at 24 h and 48 h post laser treatment. RPE flatmounts were prepared from adult RPE-cKO mice and control mice 24 h and 48 h after laser photocoagulation (24 h: n = 6 and 10 eyes; 48 h: n = 4 and 8 eyes) and stained with Ki67 (proliferative cell marker) and phalloidin (cell border). (**B**) Quantification of wound size in RPE-cKO and control groups based on phalloidin staining, which was analyzed by ImageJ with manual correction (blinded to sample groups during analysis). (**C**) Quantification of cell number within laser area (200 μm). (**D**) Quantification of Ki67-positive cell number within 500 μm. Scale bars: 20 μm. Statistical analyses were performed using Student’s t-test, p < 0.05 was considered statistically significant.

Previous studies have shown that laser photocoagulation results in RPE cell proliferation in mouse eyes during the wound healing process^73,80,81^. To investigate the involvement of primary cilia are involved in this RPE wound repair and proliferation, we used laser photocoagulation to produce an injury of consistent size to induce wound healing in our conditional RPE-cKO mice and age-matched controls. We collected RPE/choroid layer 24 h and 48 h post laser administration and visualized RPE morphology by phalloidin staining on flatmounts. At 24 h post laser treatment, no significant difference in wound size was detected in both groups. Surprisingly, the size of the wound area was significantly reduced in the RPE-cKO group compared to controls, and in the control group, resurfacing across the wound area remained incomplete at 48 h (Fig. 6A,B). In the RPE-cKO group, healing was nearly complete at this time. Compared to age-matched control groups, we found a significant reduction in the rate of primary cilia reassembly, based on Arl13b signal, in RPE-cKO mice after 48 h of laser treatment (Fig. S4, 48.70 ± 4.71% in age-matched control groups to 8.06 ± 4.95% in RPE-cKO mice). To investigate whether defective cilia promoted RPE wound healing by regulating proliferative activity, we assessed total cell number, determined by phalloidin staining and nuclei staining in the laser-treated RPE layer (diameter in 200 μm). The analysis showed a significantly increased cell number in the laser-treated area of the RPE-cKO mutant group compared to controls on both timepoints (Fig. 6C). Ki67-positive cells were found in the RPE layer of the laser-treated area and in the adjacent untreated area. We then assessed the number of Ki67-postive cells within a diameter of 500 μm, which includes most proliferating cells, and found a significant difference between RPE-cKO and control groups at 24 h post laser, indicating that defective cilia enhanced cell proliferation during healing. Ki67 positive cells were present in both groups at 48 h post laser. Taken together, these results demonstrated that defective cilia promote wound healing in mouse RPE.

## Discussion

Here we describe a novel role of primary cilia in wound repair of adult mouse RPE. We first confirmed primary cilia retraction on different mouse lines from previous reports and demonstrated that BBS4 contributes to ciliation. These data extend previous findings for RPE cilia during development^63,64^. We also found that primary cilia persist on RPE during adulthood in humans, suggesting that they continue to contribute to cellular functions after maturation. In addition, we showed that primary cilia are reassembled after injury, that they persist during the repair process, and that conditional depletion of Ift88 from RPE influences proliferation, which promotes wound healing.

Only a few studies have focused on understanding the morphology and function of primary cilia on RPE in vivo which were ignored for decades. It is known that primary cilia are essential for RPE maturation in mice and act by regulating canonical Wnt signaling^63^. Primary cilia of RPE are also known to be resorbed after maturation, which is dependent on BBSome proteins and canonical-Wnt signaling^64^, although conflicting reports have suggested that cilia in mouse RPEs are retained throughout adulthood^82^. The present study found that primary cilia levels in albino mice also decrease with increasing developmental stages, supporting the reports from Patnaik et al.^64^. In addition, we found that cilia retraction time was delayed in albino mice compared to pigmented C57BL/6 mice. This suggested to us that perhaps in an albino retina prone to potential light-induced injury processes cilia may remain and be involved in a potential repair process not easily observed in a pigmented mouse retina. A prior cilia study on albino rats also discovered primary cilia at the postnatal stage^65^. It has been shown that ciliogenesis negatively controls melanogenesis in melanocytes^83^. Alternatively, the differences in retraction time may be due to strain differences or directly related to melanin synthesis. BBS6 and BBS8 have been demonstrated as being involved in cilia disassembly and RPE maturation in mouse RPE during development^64^. Specifically, BBS8- deficiency RPE displayed phenotypic defects and affected RPE homeostasis^84^. Here, we showed a significant role of BBS4 in RPE ciliogenesis during development. Interestingly, previous reports indicate that the absence of BBS8 or BBS6 impaired both ciliation and ciliary length in mouse RPE. We did not find any changes in ciliary length in BBS4 knockout RPE. Moreover, we found that human RPEs retain stable ciliation levels at the adult age. The differences in ciliation profiles are, therefore, likely to be caused by species-specific differences in primary cilia regulation. Interestingly, we found that human RPE cells retain stable ciliation levels at adult ages. The differences in ciliation profiles are likely caused by species-specific differences in primary cilia regulation. The primary cilia in mouse RPE may be crucial in this tissue and other ocular cells in retinal development, but dispensable for cellular function in adult RPE because mice do not possess a macula. In contrast, primary cilia appear to be essential in the RPE of adult humans, and impaired in retinal disease. Therefore, mice are not likely to provide optimal models for the investigation of eye ciliopathies.

During the wound repair process, epithelial cells that surround the lesion proliferate and migrate to re-surface the wound area^60,85^. Primary cilia are postulated to undergo major alterations during wound healing^58,60,86^, and the cilia assembly/disassembly process is considered to be deeply involved in the injury response. In corneal endothelial cells, primary cilia reform rapidly at the early stage of the tissue response to mechanical injury, navigating cellular morphogenesis and patterning^72^. After wound healing is completed and morphogenesis stable, primary cilia are reabsorbed. In inner ear hair cells, for example, adult cilia regrow following trauma^87^. In kidney tubular cells, the length of primary cilia increases during renal repair^88^. Our present data have shown that cilia are rapidly reassembled in mouse RPE rapidly occurs during laser-induced wound healing and then disassembled when the repair process is completed.

Previous studies showed that primary cilia passively influence cell proliferation and the cell cycle^62,89^. Cilia disassemble when cells re-enter the cell cycle and reassemble at the G1/G0 phase of the cell cycle. It is not surprising that primary cilia negatively regulate RPE proliferation during repair. We found that depletion of Ift88 for postnatal RPE promotes wound repair rate by enhancing cell proliferation. The underlying mechanism needs to be further investigated, but the ß-catenin/Wnt pathway may be at least in part responsible, since it has been reported to be induced by laser photocoagulation in vivo^73^.

In the RPE development study, one of the technical limitations was using only one ciliary marker, Arl13b, to visualize primary cilia in the mouse and human RPE tissues. This limitation may partly explain the differences in ciliation between our data and those of other publications^64^. Moreover, we used a late-mature RPE Cre-driver, BEST1, to genetically remove Ift88 from the mouse RPE. Since BEST1-promoter expression occurs late, cilia removal may have a limited impact on RPE maturation and homeostasis.

In conclusion, this study provides insight into the role of the primary cilia in adult RPE in vivo. We showed that primary cilia reform rapidly during RPE repair which negatively regulates wound healing by inhibiting proliferation, thus providing a potential therapeutic target in RPE degenerative diseases. We also showed that human RPE continues to express primary cilia in adulthood. Together, these findings increase understanding of the role played by primary cilia in the pathogenesis of defective RPE wound repair.

## Materials and methods

### Immunolabeling reagents

Primary antibodies, source, catalog number and dilutions are as follows: anti-Arl13b mouse (Antibodies Incorporated, N295B/66, 1:2000); anti-zonula occludens-1 (ZO-1) rabbit (Invitrogen, 617300, 1:350); anti-Cre mouse (Millipore, clone 2D8, 1:400); anti-RPE65 mouse (Abcam, ab13826, 1:500); anti-Ezrin mouse (Millipore, E8897, 1:500); anti-Ki67 mouse (Cell Signaling, 9449 T, 1:500); rhodamine phalloidin (Invitrogen, R415, 1:1000). Secondary antibodies: AlexaFluor 488, 647 and 594 IgG (Life Technologies, 1:200). ProLong Gold Antifade Mount and DAPI both from Invitrogen.

### Mice

The CD-1 IGS mice were obtained from Charles River Laboratory. The Arl13b-mCherry; Centrin2-GFP mice were purchased from The Jackson Laboratory (referred to as Arl mice, stock No. 027967). This double transgenic strain expresses fluorescence mCherry and GFP to label ciliary protein (Arl13b) and basal bodies protein (Centrin2) respectively. An RPE-specific *Ift88* knockout mouse was generated by crossing the floxed *Ift88* mice (The Jackson Laboratory stock No. 022409) with BEST1-Cre transgenic mice^71^. PCR for mouse genotyping was performed using tail biopsy genomic DNA as described^90^. The primers were: BEST1-Cre, Forward: TCCGAAAAGAAAACGTTGATG; Reverse: CCCGGCAAAACAGGTAGTTA; Heterozygous animals were used as wild type controls. Animals were housed under a 12-h light/dark cycle with free access to water and food. Mice were dark-adapted overnight and euthanized under dim red light by CO_2_ inhalation. Eyes were enucleated and dissected in cold PBS, followed by fixation in freshly made 4% paraformaldehyde for 1 h at room temperature.

Eyes from BBS4 mutant mice and controls were obtained from Dr. Nicolas Berbari (Indiana University). BBS4 eyes were harvested from mutant and control wildtype pups on the day of birth (P0), fixed in 4% paraformaldehyde overnight at 4 °C and then shipped in cold PBS.

### Laser photocoagulation

Mice were anesthetized by a mixture of xylazine (0.01 mg/g) and ketamine (0.08 mg/g), followed by pupil dilation with tropicamide (Akorn, Somerset, New Jersey) and phenylephrine. A 577-nm PASCAL laser (Topcon Medical Laser System, Inc., Santa Clara, CA) assisted by a slit-lamp system was used to induce single laser lesions in mouse eyes^91^. The laser parameters were as follows: typical power of 70 mW, duration of 20 ms, aerial spot diameter of 200 μm. 6 laser spots were evenly distributed over mouse retina with at least 1 mm distance from each spot. The laser energy is absorbed by pigmentation in RPE cells and emitted as heat to the retina, resulting in both RPE and photoreceptor cell death. Success of the treatment is determined by an immediately visible retinal whitening and leakage on fluorescein angiography.

### Electroretinography (ERG)

Mouse pupils were dilated by tropicamide and phenylephrine 10 min prior to administering anesthesia as described^92^. Mice were dark-adapted for 12 h before ERG recording. ERG recording was performed with a Celeris ERG stimulator (Diagnosys LLC) under dim red light according to instructions. Briefly, mice were placed on a heating pad and artificial tears were applied to the corneal surface to prevent dryness. Two ERG electrodes were gently placed on the corneal surface and light stimulation was controlled by Espion software as described (version 6; Diagnosys)^70^. Four mice per group and 300 reads per eye were recorded. Three replications were conducted in the study.

### Optical coherence tomography (OCT) imaging

OCT imaging was performed immediately after ERG recording. A contact lens (Advanced Vision Technologies) was placed on the mouse cornea before applying artificial tears to maintain corneal moisture as described^92^. OCT images were acquired using Heidelberg Spectralis SLO/OCT system (Heidelberg Engineering, Germany). The retina scans were obtained and aligned with images centered around the optic nerve head. Photoreceptor layer thickness was measured by double-blinded masking of samples and averaged by Heidelberg software. The mean of the photoreceptor thickness in RPE-cKO mice was compared to heterozygous mice as a control.

### Immunofluorescence staining and flatmount

For RPE flatmount experiments, mice were dark-adapted for 6 h before their eyes were enucleated. Retina tissue and anterior segment (cornea, iris and ciliary body, and lens) were immediately removed in cold PBS under dim red light. RPE/choroid layers were then immersed in 4% PFA fixation for 1 h at room temperature. Fixed tissues were washed with PBS 3 times and incubated for 1 h in blocking buffer containing 10% goat serum, 0.3% Triton X-100 at room temperature. After blocking, RPE/choroid layers were incubated with primary antibodies at 4 °C overnight. Following washes in PBS, tissues were incubated in the blocking buffer containing secondary antibodies for 1 h at room temperature. Nuclei were stained with DAPI nuclear stain 405. Eight radial cuts were made from the edge of the eyecup to the equator before being mounted with ProLong Gold (Life Technologies) on coverslips. Confocal imaging was performed with a Zeiss LSM880 confocal microscope.

### Human tissue and tissue processing

De-identified tissue samples of healthy human and age-related macular degeneration donor eye were obtained from Stanford University’s Department of Pathology, Palo Alto, CA. Eye was obtained within 48 h of death and stored in a moist chamber at 4 °C after enucleation. The cornea, iris, lens, and vitreous were removed through a coronal incision made 3 mm posterior to the limbus. The globes were fixed in 4% PFA overnight and washed in 1 × PBS at 4 °C. The eye was from a patient carrying no posterior segment pathology. A layer of RPE approximately 6–8 mm^2^ was dissociated from the retina and choroid before flattening using micro-dissection technique.

### iDiSCO

Fixed human RPE tissue was washed in PBS for 1 h, then in 50% methanol (in PBS) for 1 h, 80% methanol for 1 h, and 100% methanol for 1 h twice. Samples were then bleached with 5% H_2_O_2_ in 20% DMSO/methanol at 4 °C overnight. After bleaching, samples were washed in methanol for 1 h, then in 20% DMSO/methanol for 1 h twice, then in 80% methanol for 1 h, 50% methanol for 1 h, PBS for 1 h twice, and finally in PBS/0.2% Triton X-100 for 1 h twice before further staining procedures^69^.

### Primary cilia, wound area measurement and fluorescence intensity quantification

A primary cilium was counted based on Arl13b signal in Figs. 1 and 2: to distinguish from the nonspecific background, a continuous Arl13b-positive signal with a clear, linear structure was count as a primary cilium. Specifically, RPE flatmounts were captured using a Zeiss LSM 880 confocal microscope at 63× with 0.6× optical zoom (223 × 223 μm^2^). The images were captured with Z-stack (0.3 μm interval) for better characterization of primary cilia from nonspecific staining. In Fig. 5, the primary cilia were determined by two primary ciliary markers (Arl13b and centrin2 signal).

For wound size measurement, we analyzed the reepithelialization on lasered RPE flatmounts of RPE-cKO and control mice at 24 h and 48 h after laser treatment (blinded to sample groups). For this purpose, we utilized anti ZO-1 antibody or phalloidin to visualize cell borders. The size of the un-epithelized area was measured by FIJI ImageJ software (National Institutes of Health Bethesda, MD, USA) with manual assist. Fluorescence intensity quantification for RPE-65 and ezrin staining was calculated by averaging pixel intensity using Zeiss software in accordance with recommended guidelines.

### Statistics

All statistical analysis was performed using Graphpad8 (Prism) software. Results are expressed as mean values ± SEM. Statistical analysis was processed by unpaired t-test for comparison of two groups. P-values below 0.05 were determined as statistically significant.

### Study approval

Our work on human samples was conducted in accordance with the principles of the Declaration of Helsinki for medical research involving human subjects. The study was approved by the Institutional Review Board (IRB) at Stanford University. This study is reported in accordance with ARRIVE guidelines. Prior signed written informed consent was received from authorized legal representatives for the use of samples for research purposes prior. All procedures related to animals were in compliance with the guidelines of the Association for Research in Vision and Ophthalmology Statement for the Use of Animals in Ophthalmic and Vision Research, and all animal experiments were approved by the Institutional Animal Care and Use Committee (IACUC) of Stanford University School of Medicine. All procedures for BBS4 mice were approved by the IACUC at Indiana University-Purdue University Indianapolis.

## Supplementary Information

Supplementary Legends.

Supplementary Figures.

## Data Availability

The datasets used and/or analyzed during the current study available from the corresponding author on reasonable request.
