# Supplementary material for: Cilia-associated wound repair mediated by IFT88 in retinal pigment epithelium
Source: Sci Rep. 2023 May 21;13:8205. doi: 10.1038/s41598-023-35099-3 (PMC10200793; doi:10.1038/s41598-023-35099-3)
Supplement: Supplementary file 1 — Supplementary Legends. [file 41598_2023_35099_MOESM1_ESM.docx]

**Supplemental figure 1. Examples of Bi-ciliated RPEs in CD1 mice.** Representative images of ZO-1 (red) and Arl13b (green) antibodies in RPE flatmounts of P0 or P7 CD1 mice (n=3, 200-300 ciliated RPEs were counted per timepoint). Scale bars: 20 μm.

**Supplemental figure 2. Description of RPE-cKO mice.** (**A**) Immunofluorescence analysis of ZO-1 (red) and Ezrin (green) antibody in RPE flatmounts of RPE-cKO and control mice. Expression level was quantified by analyzing the integrated fluorescence intensity. (**B**) Electroretinogram (ERG) recordings for RPE-cKO and control mice. Light-adapted ERG showing the cone photoreceptor function. n=4 mice. Scale bars: 20 μm. n=3 animals in 2-month RPE-cKO group; n=5 animals in 2-month control group; n=5 animals in 6-month RPE-cKO group; n=3 animals in 6-month control. Statistical analyses were performed using one-way ANOVA, p<0.05 was considered statistically significant.

**Supplemental figure 3**. Regional hypopigmentation in RPE-cKO mice. Few examples of RPE flatmounts from RPE-cKO mice displaying patchy/regional hypopigmentation in the peripheral area of RPE, pointed by arrows. Scale bars: 300 μm.

**Supplemental figure 4**. Decreased reassembly rate of primary cilia in RPE-cKO mice. (A-B) Immunofluorescence analysis of ZO-1 (red), Arl13b (green) and Cre (green) antibodies in RPE flatmounts of RPE-cKO and control mice at 48 hrs post laser treatment. (A’-B’) High magnification images of A and B. (C) Quantification of ciliation in cells that adjacent to the laser spots (2-3 rows, total 150 cells/group were counted). Scale bars: 20 μm. Statistical analysis was performed using Student’s t-test, p<0.05 was considered statistically significant.
