# Supplementary figures and images for: Cilia-associated wound repair mediated by IFT88 in retinal pigment epithelium
Source: Sci Rep. 2023 May 21;13:8205. doi: 10.1038/s41598-023-35099-3 (PMC10200793; doi:10.1038/s41598-023-35099-3)

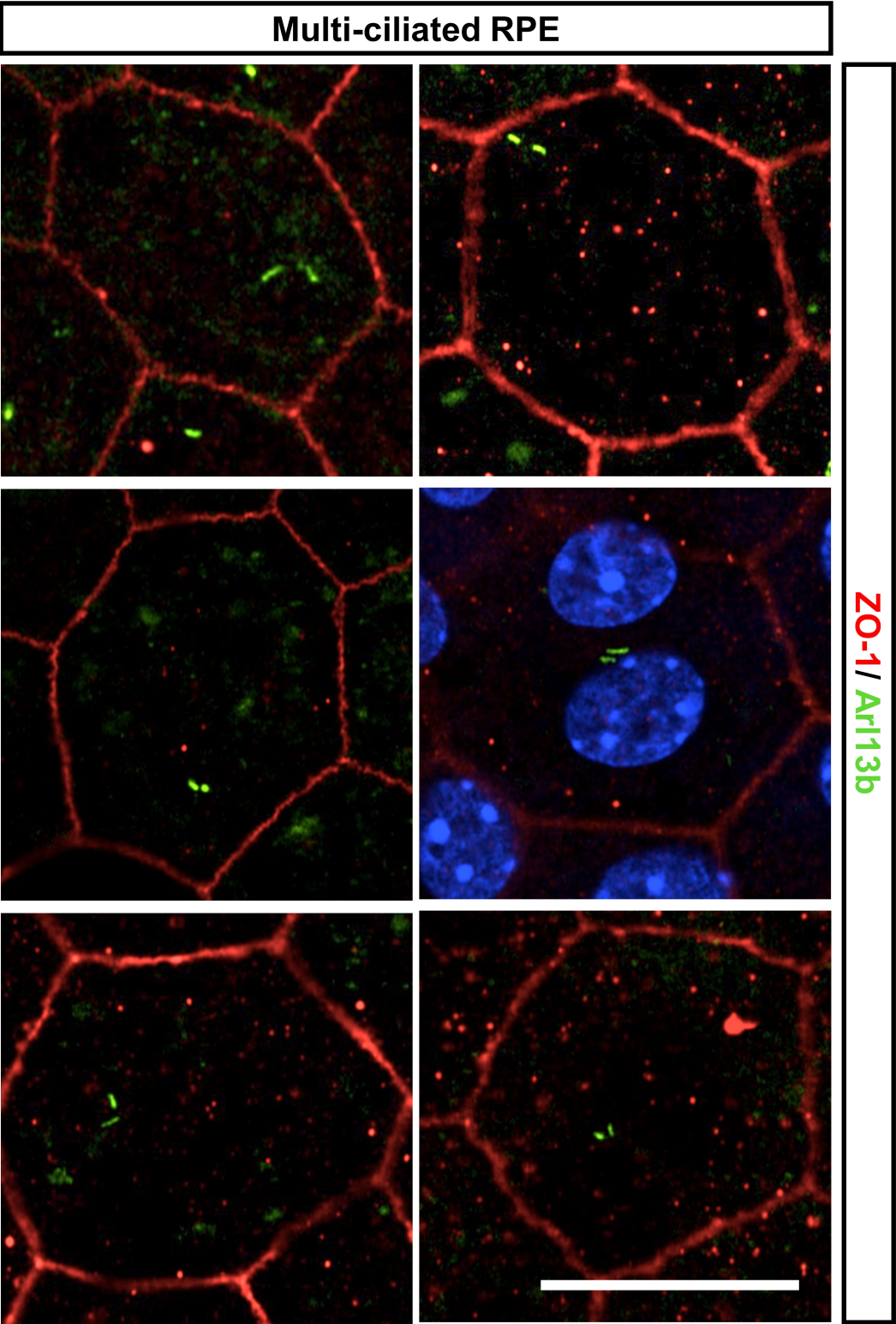

Supplemental Figure 1

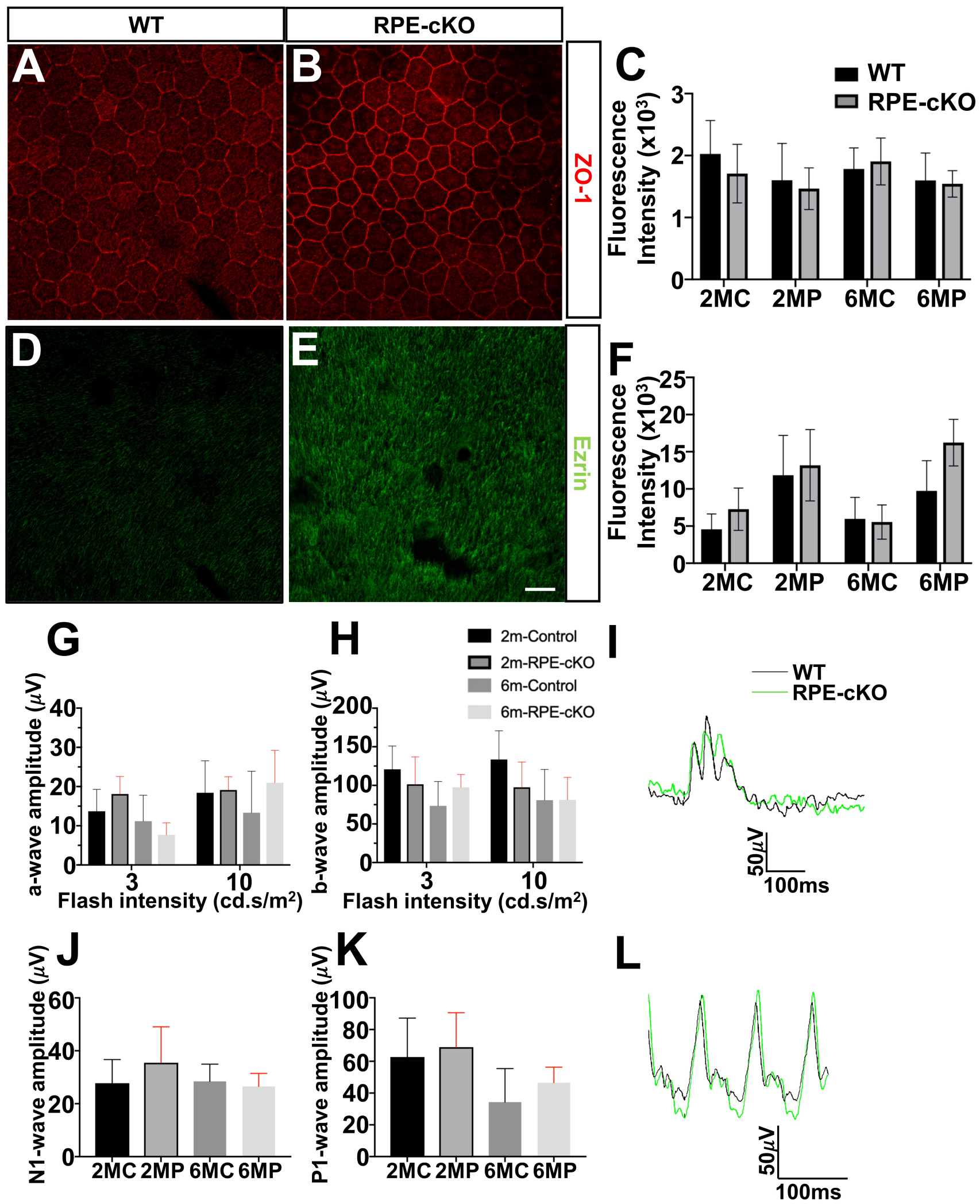

Supplemental Figure 2

RPE-cKO

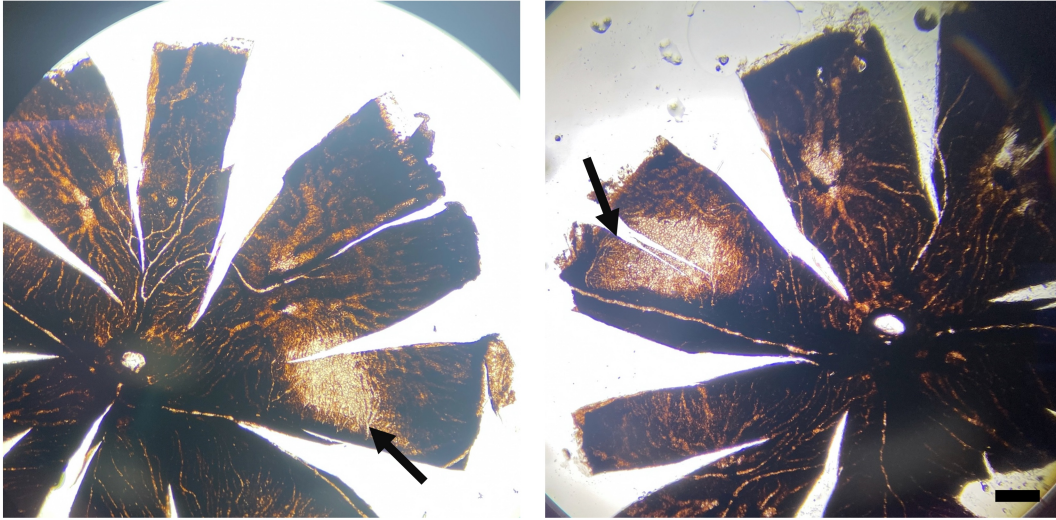

**Supplemental Figure 3**

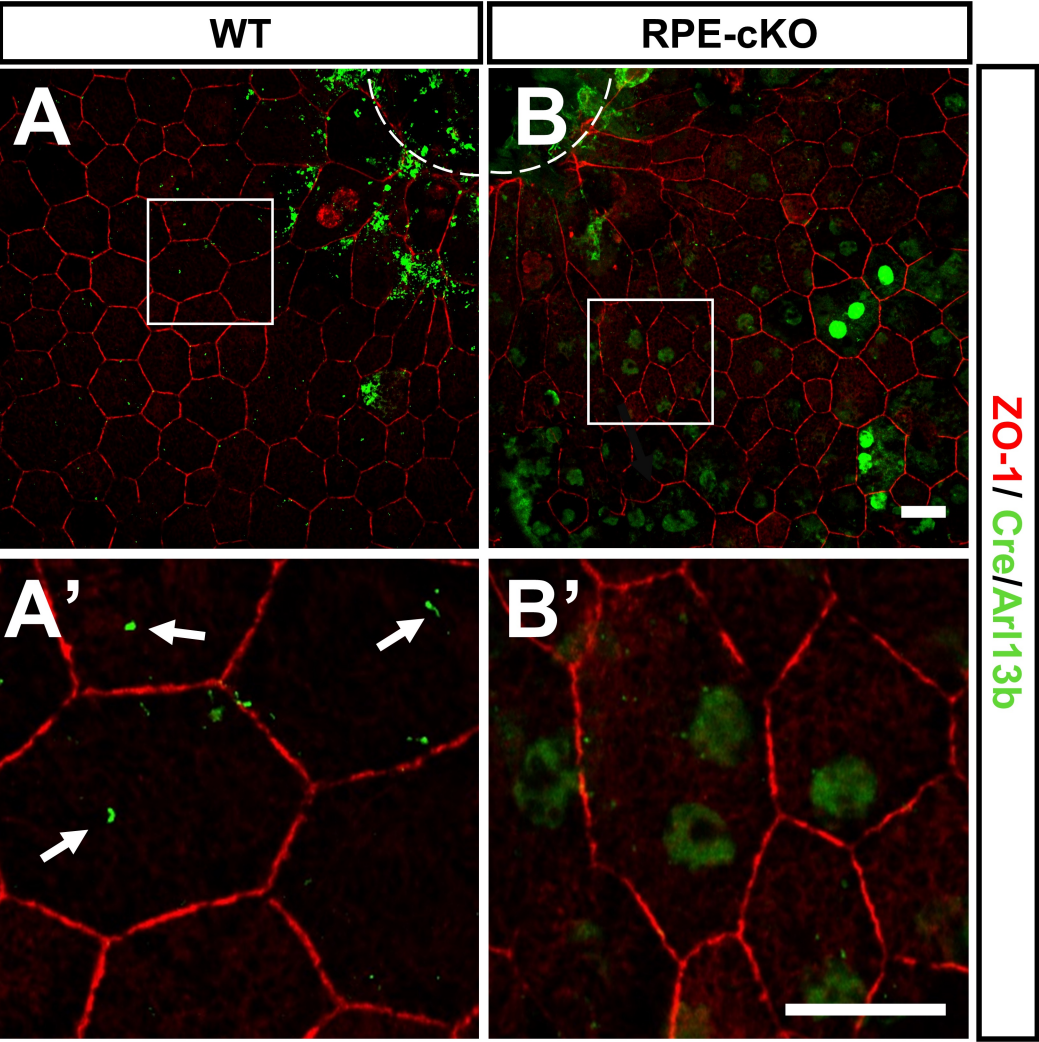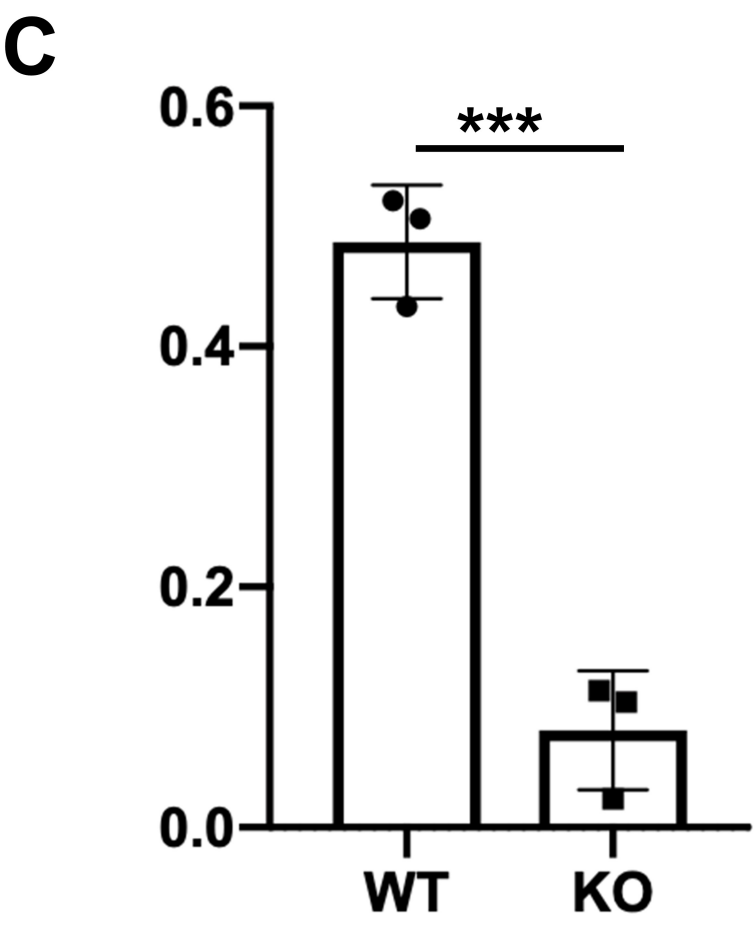

Supplemental Figure 4

Supplement: Supplementary file 2 — Supplementary Figures. [file 41598_2023_35099_MOESM2_ESM.pdf]
